# Cerebral sinovenous thrombosis and asparaginase re‐exposure in patients aged 1–45 years with acute lymphoblastic leukaemia: A NOPHO ALL2008 study

**DOI:** 10.1002/jha2.484

**Published:** 2022-06-24

**Authors:** Mette Tiedemann Skipper, Cecilie Utke Rank, Kirsten Brunsvig Jarvis, Line Stensig Lynggaard, Liv Andrés‐Jensen, Petter Quist‐Paulsen, Ruta Semaskeviciene, Helene Hallböök, Ulla Waitiovaara‐Kautto, Susanna Ranta, Sonata Trakymiene, Jonas Abrahamsson, Pasi Huttunen, Birgitte Klug Albertsen, Kjeld Schmiegelow, Ruta Tuckuviene

**Affiliations:** ^1^ Department of Paediatrics and Adolescent Medicine Aarhus University Hospital Aarhus Denmark; ^2^ Department of Clinical Medicine Aarhus University Aarhus Denmark; ^3^ Department of Paediatrics and Adolescent Medicine Rigshospitalet University Hospital Copenhagen Denmark; ^4^ Department of Paediatric Haematology and Oncology Oslo University Hospital Oslo Norway; ^5^ Department of Haematology Trondheim University Hospital Trondheim Norway; ^6^ Oncology and Transfusion Medicine Centre Vilnius University Hospital Santaros Klinikos Vilnius Lithuania; ^7^ Department of Medical Sciences Uppsala University Uppsala Sweden; ^8^ Department of Haematology Comprehensive Cancer Centre Helsinki University Hospital and University of Helsinki Helsinki Finland; ^9^ Astrid Lindgren Children's Hospital Karolinska University Hospital and Childhood Cancer Research Unit Department of Women's and Children's Health Karolinska Institute Stockholm Sweden; ^10^ Clinic of Children's Diseases Faculty of Medicine Vilnius University Vilnius University Hospital Santaros Klinikos Vilnius Lithuania; ^11^ Department of Paediatrics Institution of Clinical Science Sahlgrenska Academy University of Gothenburg Gothenburg Sweden; ^12^ Department of Paediatric Haematology Oncology and SCT New Children's Hospital Helsinki University Hospital Helsinki Finland

**Keywords:** acute leukaemia, chemotherapy, childhood leukaemia, late effects of therapy, thrombosis

## Abstract

Cerebral sinovenous thrombosis (CSVT) is a serious complication during asparaginase therapy in patients with acute lymphoblastic leukaemia (ALL). We identified 46 patients with CSVT among 2651 patients (1‒45 years) treated according to the Nordic Society of Paediatric Haematology and Oncology (NOPHO) ALL2008 protocol between 2008 and 2018. CSVT cases were prospectively registered in the NOPHO database with retrospective updates. We examined the frequency of asparaginase re‐exposure after CSVT, potential factors associated with asparaginase truncation, and sequelae after CSVT. This work was supported by the Danish Cancer Society and the Danish Childhood Cancer Foundation. The 2.5‐year cumulative incidence of CSVT was 1.9% (95% confidence interval 1.4%–2.5%). The majority of patients (74%, *n* = 31) were re‐exposed to asparaginase (with low‐molecular‐weight heparin coverage), one of whom had a second CSVT, without neurological sequelae. Patients re‐exposed to asparaginase were earlier in ALL treatment and lacked more asparaginase doses than non‐re‐exposed patients at CSVT diagnosis (median 50 vs. 81 days, *p* = 0.03; mean 11.2 vs. 8.4 asparaginase doses, *p* = 0.04). No other examined factors had an impact on asparaginase re‐exposure. At the last follow‐up (median 4.5 years after CSVT), 61% of patients had normal neurological status, and 57% had complete recanalisation of CSVT, with no significant difference between patients re‐exposed and non‐re‐exposed to asparaginase. Our results indicate that re‐exposure to asparaginase is safe after CSVT during anticoagulation.

## 1 INTRODUCTION

Cerebral sinovenous thrombosis (CSVT) is a potentially fatal complication seen in 1%–3% of patients with acute lymphoblastic leukaemia (ALL) during treatment [1, 2]. Multiple factors, including the leukaemia itself, asparaginase (ASP), corticosteroids, infection and high body mass index (BMI), adversely affect the haemostatic balance and increase the risk of thromboembolism (TE) [3, 4, 5, 6, 7, 8, 9]. If CSVT occurs, the anticipated risk of progression of CSVT or re‐thrombosis influences the decision to re‐expose patients to ASP; thus, ASP is often truncated [10, 11, 12, 13, 14, 15]. Premature discontinuation of ASP has been associated with an increased risk of leukaemic relapse [4, 14, 16, 17]. To date, no studies have examined the factors that may impact the decision to re‐expose patients to ASP after CSVT, and only a few studies have investigated the potential risks of re‐exposure to ASP [13].

Our primary aim was to determine the frequency of ASP re‐exposure after CSVT in patients with ALL. The secondary aims were to examine (a) clinical characteristics associated with ASP re‐exposure, (b) CSVT‐related sequelae, and (c) the modification of ALL treatment following CSVT.

## 2 SUBJECTS AND METHODS

### 2.1 Design and population

This population‐based, observational study was conducted using the RECORD statement [18]. The study population included children and adults aged 1–45 years at the time of ALL diagnosis who were treated according to the Nordic Society of Paediatric Haematology and Oncology (NOPHO) ALL2008 protocol between July 2008 and October 2018. Baseline clinical characteristics and treatment data were retrieved from the NOPHO register along with predefined prospectively registered toxicities, including TE [19]. Patients with symptomatic CSVT were compared with patients undergoing the same ALL treatment without TE (the comparison population). Detailed clinical data were collected retrospectively from medical charts by local investigators between March and December 2020. Data were entered into standardised registration forms corresponding to three timepoints: (a) at CSVT diagnosis, (b) before ASP re‐exposure (in patients re‐exposed to ASP), and (c) at the last follow‐up. Of 46 CSVT cases, 31 have previously been reported as part of a NOPHO study, and 20 cases have previously been reported in a smaller study on CSVT without information on neurologic sequelae [8, 20].

### 2.2 The NOPHO ALL2008 protocol

The NOPHO ALL2008 treatment protocol has previously been described in detail [21]. Briefly, the protocol includes a 4‐week induction phase including high‐dose steroids and ASP treatment from day 30 [20, 21]. All non‐high‐risk patients received five doses of pegylated ASP (PEG‐ASP) 1000 IU/m^2^ intramuscularly at 2‐week intervals during consolidation therapy and were randomised to receive either (a) 10 additional PEG‐ASP doses or (b) three additional PEG‐ASP doses [22]. High‐risk patients received one dose of ASP in each block during seven to nine blocks of chemotherapy unless the patient was eligible for bone marrow transplantation. For further details on the treatment protocol and definition of central nervous system (CNS) leukaemia, see Supporting Information. Asparaginase enzyme activity (AEA) was measured systematically in all children as part of the treatment protocol.

### 2.3 Definition of clinical characteristics

BMI was reported as *Z*‐scores of a Danish normal reference population [23]. Major and minor bleeding during antithrombotic treatment were defined according to the definition by Schulman and Kearon [24]. Thrombophilia screening was recommended if TE occurred and was conducted at local certified coagulation laboratories. Exposure to ASP after CSVT was denoted as re‐exposure, regardless of ASP administration prior to CSVT. CSVT was considered to be associated with ASP if CSVT was diagnosed within 18 days following the last administration of ASP [22]. The total number of ASP doses missed was calculated as the number of planned doses for a single patient minus the doses given.

The neurological status in patients with CSVT was quantified by the local physician using the modified Rankin Scale (mRS) [25] at three timepoints: (a) at CSVT diagnosis, (b) before ASP re‐exposure (in patients re‐exposed to ASP), and (c) at last follow‐up. Neurological status was classified as no symptoms (mRS 0), no significant disability despite symptoms (mRS 1), slight disability (mRS 2), moderate disability (mRS 3), moderately severe disability (mRS 4), severe disability (mRS 5) and death (mRS 6) [25, 26]. Detailed descriptions of diagnostic imaging were used to study the extent of the thrombosis and brain lesion at (a) CSVT diagnosis, (b) before re‐exposure, and (c) the time of the last follow‐up scan. Venous infarction was defined as necrotic tissue visible on imaging in relation to the thrombosis and described by the local radiologist. The extent and number of thromboses were scored according to the CSVT score developed by Zubkov et al. [27], assigning one point for each sinus involved and one point for each third of the superior sagittal sinus. Recanalisation of veins before re‐exposure and at the last follow‐up was quantified according to Qureshi [28] and classified as partial recanalisation of one or more occluded veins (grade I), complete recanalisation of one sinus but persistent occlusion of the other sinuses (grade II) and complete recanalisation (grade III).

### 2.4 Statistics

Patients were followed from the time of ALL diagnosis until CSVT, death, second malignant neoplasm, leukaemic relapse, or loss to follow‐up, which ever came first (Table 1). CSVT patients were further followed until death or the last follow‐up date at the local centres (Tables 2 and 3). CSVT patients were excluded from the re‐exposure/non‐re‐exposure analyses if (a) the patient had already received all doses of ASP prior to the CSVT diagnosis and (b) the patient discontinued ALL treatment after CSVT diagnosis due to poor clinical condition followed by death or died sooner than the patient was scheduled to receive the next ASP dose (2‐ or 6‐week interval depending on randomisation) (a priori definition). The cumulative incidence of CSVT was estimated using the Aalen‐Johansen method considering relapse, death and second malignant neoplasm as competing events. We reported continuous and normally distributed data as the mean and standard deviation (SD). Differences between groups were estimated using Student's *t*‐test. Non‐normally distributed data were reported as the median and interquartile range (IQR) and were compared using Wilcoxon's rank‐sum (Mann–Whitney) test. Categorical data were presented as the number of patients (with percent) and analysed by applying a chi‐squared test, and for samples including less than five observations in any group, Fisher's exact test was applied. We calculated the incidence of bleeding after CSVT as incidence rates during antithrombotic treatment and tested for differences between groups by applying a mid *p*‐adjustment test to explore incidence rate differences. Levels of AEA were analysed as previously described [29] and are provided in Supporting Information. A two‐sided *p*‐value <0.05 was considered statistically significant. All statistics were performed in Stata version 16.1, and graphs were generated in GraphPad Prism 9.1.2. Data are available upon request.

**TABLE 1:** 2.5‐year cumulative incidence of cerebral sinovenous thrombosis (CSVT)

	*N* (%)	2.5‐year cumulative incidence of CSVT (%)	95% CI	95% CI of difference (%)**^a^**	*p*‐Value
All patients^b^	2472	1.87	1.39–2.46	–	–
Sex					
Female	1404 (57)	1.78	1.11–2.72		0.78
Male	1068 (43)	1.94	1.31–2.76	−0.92 to 1.23	
Age at ALL diagnosis (years)					
Age group: 1–9.9	1636 (67)	1.23	0.78–1.85		
Age group: 10–17.9	423 (85)	3.8	2.26–5.94	0.67–4.48	0.01^c^
Age group: 18—45	373 (15)	2.74	1.4–4.81	−0.24 to 3.24	0.01^d^
BMI at ALL diagnosis: *Z*‐score**^e^**					
≤ −2SD	128 (5)	3.14	1.03–7.28		0.12
> −2SD to < +2SD	2099 (87)	1.63	1.15–2.24	−4.58 to 1.56	
≥ +2SD	176 (7)	4.57	2.14–8.39	−2.89 to 5.75	
Immunophenotype					
B cell	2058 (85)	1.80	1.29–2.45		0.45
T cell	374 (15)	2.47	1.22–4.47	−1.02 to 2.31	
CNS status at ALL diagnosis					
CNS1**^f^**	2106 (86)	1.71	1.23–2.35		0.19
CNS2/CNS3**^f^**	337 (14)	2.97	1.52–5.21	−0.64 to 3.16	
WBC at ALL diagnosis					
<100 × 10^9^/L	2277 (94)	1.98	1.47–2.62		0.11
≥100 × 10^9^/L	139 (6)	0.76	0.07–3.80	−0.3 to 2.78	
Enlarged lymph nodes ≥3 cm					
No	2134 (92)	1.88	1.37–2.53		0.31
Yes	187 (8)	3.24	1.34–6.54	−1.25 to 3.95	
Mediastinal mass					
No	2213 (92)	1.77	1.28–2.39		0.25
Yes	183 (8)	3.31	1.37–6.69	−1.11 to 4.18	
High‐risk stratification, day 29					
No	1973 (80)	1.93	1.39–2.61		0.98
Yes	426 (17)	1.93	0.91–3.63	−1.45 to 1.42	

Abbreviations: 95% CI, 95% confidence interval; ALL, acute lymphoblastic leukaemia; BMI, body mass index; MRI, magnetic resonance imaging; SD, standard deviation; WBC, white blood count.

^a^ 95% CI of difference in cumulative incidence between groups.

^b^ Patients with CSVT (*n* = 46) among ALL patients 1–45 years of age (*n* = 2472) treated according to the Nordic Society of Paediatric Haematology and Oncology (NOPHO) ALL2008 protocol; *n*: number of patients.

^c^ Test of heterogeneity of the three age groups.

^d^ A test for trend.

^e^ BMI *Z*‐scores of a Danish normal reference population [23].

^f^ CNS1: no blasts in cerebrospinal fluid on cytospin and no other signs of CNS leukaemia, CNS2: 0–5 cells/ml cerebrospinal fluid with blasts on cytospin and no other signs of CNS leukaemia), CNS3: >5 cells/ml cerebrospinal fluid with blasts on cytospin, cranial nerve palsy, intracranial ‘leukaemic’ mass on MRI, eye involvement confirmed by MRI, or a biopsy to reflect ALL.

**TABLE 2:** Demographics and clinical characteristics of cerebral sinovenous thrombosis (CSVT) patients with acute lymphoblastic leukaemia (ALL)

	Total (*n* = 46)		Re‐exposed to ASP^a^ (*n* = 31)		Non‐re‐exposed to ASP^a^ (*n* = 11)		
	*n*	%	*n*	%	*n*	%	*p*‐Value^a^
All CSVT patients	46	100	31	74	11	26	
Age at CSVT (years), median, IQR	(10.9)	(4.9–17.3)	(12.5)	(5.4–18.1)	(10.2)	(4.9–23.1)	0.83
Age group: 1–9.9 years	20	43	12	39	5	45	0.91
Age group: 10–17.9 years	15	33	11	35	3	27	
Age group: 18–45 years	11	24	8	26	3	27	
Sex, male	27	59	17	55	8	73	0.30
BMI *Z*‐scores, mean, SD	(0.51)	(1.67)	(0.31)	(1.76)	(0.93)	(1.4)	0.24
≤ −2SD	4	9	4	13	0		0.30
> −2SD to < +2SD	34	74	23	74	8	73	
≥ +2SD	8	17	4	13	3	27	
Down syndrome (trisomy 21)	2		1		1		
Immunophenotype: T cell	9	20	4	13	4	36	0.09
CNS status at diagnosis: CNS2/CNS3^b^	10	22	7	23	1	9	0.33
High‐risk stratification, day 29	9	20	5	16	3	27	0.42
Infection at CSVT diagnosis	11	24%	5	16%	4	36%	0.16
Days from ALL diagnosis to CSVT, median, IQR^c^	(53)	(34–91)	(50)	(32–76)	(81)	(51–149)	0.03
ASP naïve at CSVT diagnosis	8	17	6	19	1	9	0.43
CSVT within 18 days of last ASP dosage	33	87	23	92	7	70	0.09
Number of ASP doses administered at CSVT diagnosis, mean, SD	(2.7)	(2.8)	(2.1)	(2.1)	(4.4)	(3.7)	0.02
Number of missing ASP doses at CSVT diagnosis, mean, SD^d^	(10.4)	(4.0)	(11.2)	(3.4)	(8.4)	(4.8)	0.04
Date of CSVT diagnosis							
2008/12–2011/04	16	35	9	56	7	44	0.18
2011/05–2015/04	15	33	9	60	6	40	
2015/05–2018/06	15	33	13	87	2	13	

*Note*: *n* denotes the number of patients in the exposure group. *p*‐Value: test of no difference between re‐exposed and non‐re‐exposed.

Abbreviations: ASP, asparaginase; BMI, body mass index; IQR, interquartile range; MRI, magnetic resonance imaging; SD, standard deviation.

^a^ *n* = 42, excluded in the analysis: death shortly after diagnosis (*n* = 3) and already received all ASP doses at CSVT diagnosis (*n* = 1), *p*‐value: simple statistical test of differences between re‐exposed and non‐re‐exposed (no adjustments were applied because of the small sample size).

^b^ CNS2: 0–5 cells/ml cerebrospinal fluid with blasts on cytospin and no other signs of CNS leukaemia, CNS3: >5 cells/ml cerebrospinal fluid with blasts on cytospin, cranial nerve palsy, intracranial ‘leukaemic’ mass on MRI, eye involvement confirmed by MRI, or a biopsy to reflect ALL.

^c^ A histogram (Figure S1) of the number of days is provided in Supporting Information.

^d^ Planned doses of ASP were assumed to be 15 and eight doses for patients treated according to standard‐risk and intermediate‐risk stratification, depending on randomisation [22]. For patients treated according to high‐risk stratification, the number of planned doses was scrutinised for each patient at the time of CSVT diagnosis.

**TABLE 3:** Clinical characteristics, treatment and outcome

			Re‐exposure analyses (*n* = 42)^a^				
	Patients with CSVT (*n* = 46)		Re‐exposed to ASP (*n* = 31)		Non‐re‐exposed to ASP (*n* = 11)		
	*n*/*N*	%	*n*/*N*	%	*n*/*N*	%	*p‐*Value
Neurological status at diagnosis							
Seizures	17/43	40	14/29	48	2/11	18	0.08
Affected consciousness	13/45	29	8/30	27	3/11	27	0.97
Visual disturbance and/or papilloedema	4/34	12	1/21	5	3/11	27	0.07
Headache	27/43	63	17/29	59	8/11	72	0.41
Nausea/vomiting	12/43	28	10/29	34	2/11	18	0.32
Motor difficulties	16/43	37	11/29	38	4/11	36	0.93
Modified Rankin Scale, median, IQR	(2)	(1–4)	(2)	(1–2.75)	(3)	(1.8–3.25)	0.14
Imaging at diagnosis							
Parenchymal lesions	16/36	44	11/23	48	3/10	30	0.34
Bilateral lesions	7/34	21	4/21	19	1/10	10	0.52
Oedema	8/35	23	6/23	26	0		0.09
Haemorrhage	11/36	31	6/23	26	3/10	30	0.82
≥1 venous infarction	8/35	23	6/22	27	1/10	10	0.27
CSVT score							
1 point	11/39	28	8/27	30	2/9	22	0.13
2 points	7/39	18	7/27	26	0		
3–4 points	15/39	38	10/27	37	4/9	44	
>4 points	6/39	15	2/27	7	3/9	33	
Treatment							
Days on any antithrombotic treatment, median, IQR	(232)	(183–403)	(240)	(184–408)	(280)	(175–662)	0.72
Truncation of ASP after CSVT							
≥1 ASP dose omitted	37/44	84	25/31	81	11/11	100	0.12
Number of ASP doses omitted, median, IQR	(2.5)	(1–6.75)	(2)	(1–5)	(6)	(4–8)	<0.01
≥1 intrathecal treatment omitted	14/43	33	9/30	30	4/11	36	0.7
Outcome at follow‐up							
Follow‐up (years), median, IQR^b^	(4.5)	(2.8–7.4)	(4.6)	(2.8–6.8)	(7.9)	(3.3–9.5)	0.43
Major bleeding during antithrombotic treatment, no. (IR)	4/46	(0.10)	1/31	(0.039)	1/11	(0.079)	0.66
Epilepsy treatment ongoing	4/41	10	4/30	13	0		0.56
Visual disturbance	1/41	2	1/31	3	0		1
Headache	3/40	8	2/29	7	1/10	10	1
Motor difficulties	3/41	7	2/30	7	1/10	10	1
Modified Rankin Scale, median, IQR	(0)	(0–1)	(0)	(0–1)	(0)	(0–2)	0.57
Imaging at follow‐up							
Recanalisation							
No recanalisation	1/35	3	1/23	4	0		0.48
Grade I: partial	10/35	29	6/23	26	3/10	30	
Grade II: complete of ≥1	4/35	11	1/23	4	2/10	20	
Grade III: complete of all	20/35	57	15/23	65	5/10	50	
CSVT score at follow‐up							
No thrombosis, 0 points	19/31	61	14/21	67	5/9	56	0.43
1 point	9/31	29	6/21	29	2/9	22	
2 points	2/31	6	1/21	5	1/9	11	
3–4 points	1/31	3	0		1/9	11	

Abbreviations: ASP, asparaginase; CSVT, cerebral sinovenous thrombosis; IQR, interquartile range; IR, incidence rate.

^a^ Four patients were excluded from the analysis: death shortly after diagnosis (*n* = 3) and already received all ASP doses (*n* = 1), *p*‐value: simple statistical test of differences between re‐exposed and non‐re‐exposed (no adjustments were applied because of the small sample size), *n*: number of patients with CSVT, *N*: number of patients with CSVT and available data, IR is the incidence rate per year at risk. Motor difficulties: trouble walking, impaired function of arms/hands. Infarction is defined as dead tissue visible in the scan in an area related to cerebral thrombosis. Major bleeding was defined according to Schulman and Kearon [24].

^b^ The maximal follow‐up defined as the last day of follow‐up at the local centre. Recanalisation was defined according to Qureshi [28].

## 3 RESULTS

### 3.1 Patient demographics

**FIGURE 1:**
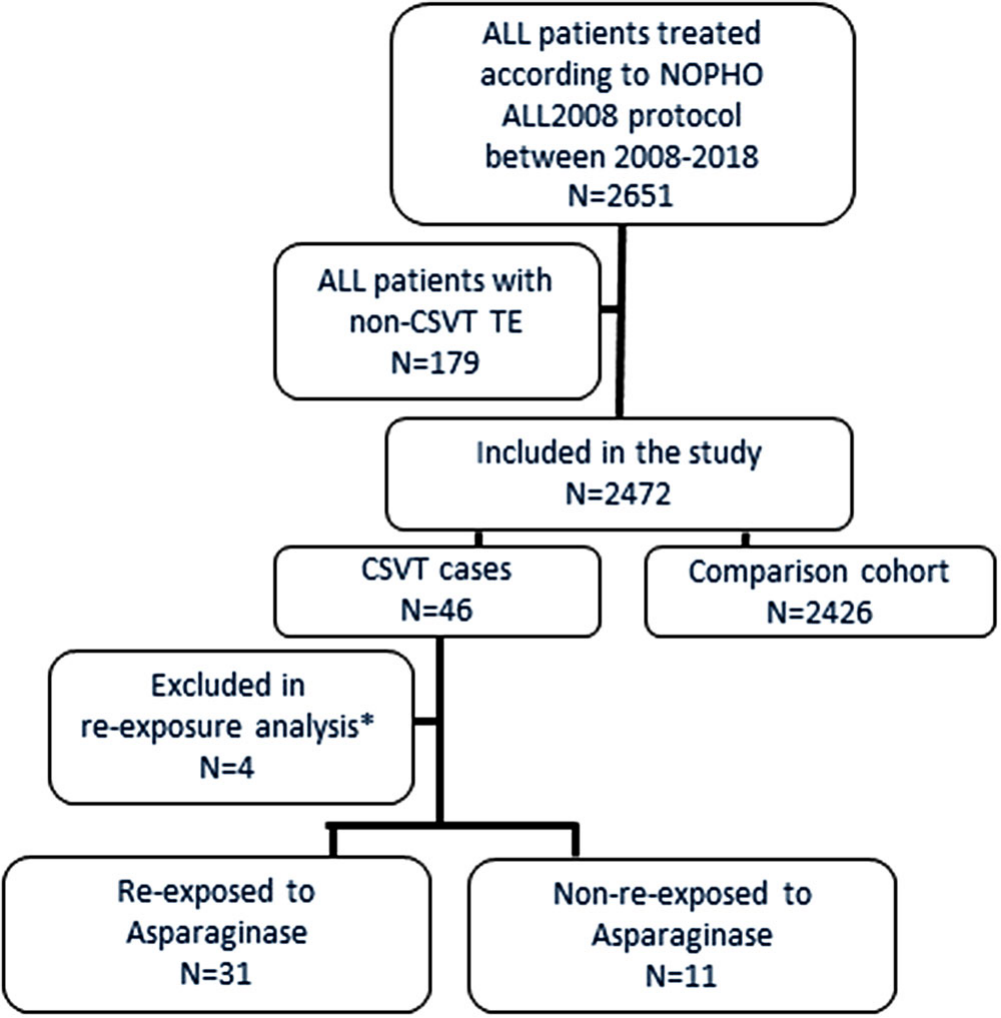
Flowchart. *Three patients died and did not receive acute lymphoblastic leukaemia (ALL) treatment after cerebral sinovenous thrombosis (CSVT); one had received all asparaginase doses prior to CSVT

A total of 2651 patients aged 1–45 years at ALL diagnosis were treated according to the NOPHO ALL2008 protocol between 2008 and 2018. Forty‐six patients were registered with CSVT, and detailed data were available in all cases. The comparison population included 2426 patients (Figure 1) who were followed for a mean of 2.3 years from ALL diagnosis (SD: 0.52). The cumulative incidence of first‐time CSVT during ALL therapy was 1.9% (95% CI: 1.4%–2.5%), which was highest among adolescents (Table 1). No other clinical characteristics were associated with a higher 2.5‐year cumulative incidence of CSVT (Table 1). Three of 46 CSVT patients died shortly after CSVT and did not receive any ALL treatment after CSVT diagnosis (CSVT diagnosed by autopsy [*n* = 1], death due to typhlitis and intestinal perforation 4 days after CSVT diagnosis [*n* = 1], death due to cerebral bleeding and herniation 11 days after CSVT diagnosis [*n* = 1]).

### 3.2 Clinical characteristics of the CSVT population

**FIGURE 2:**
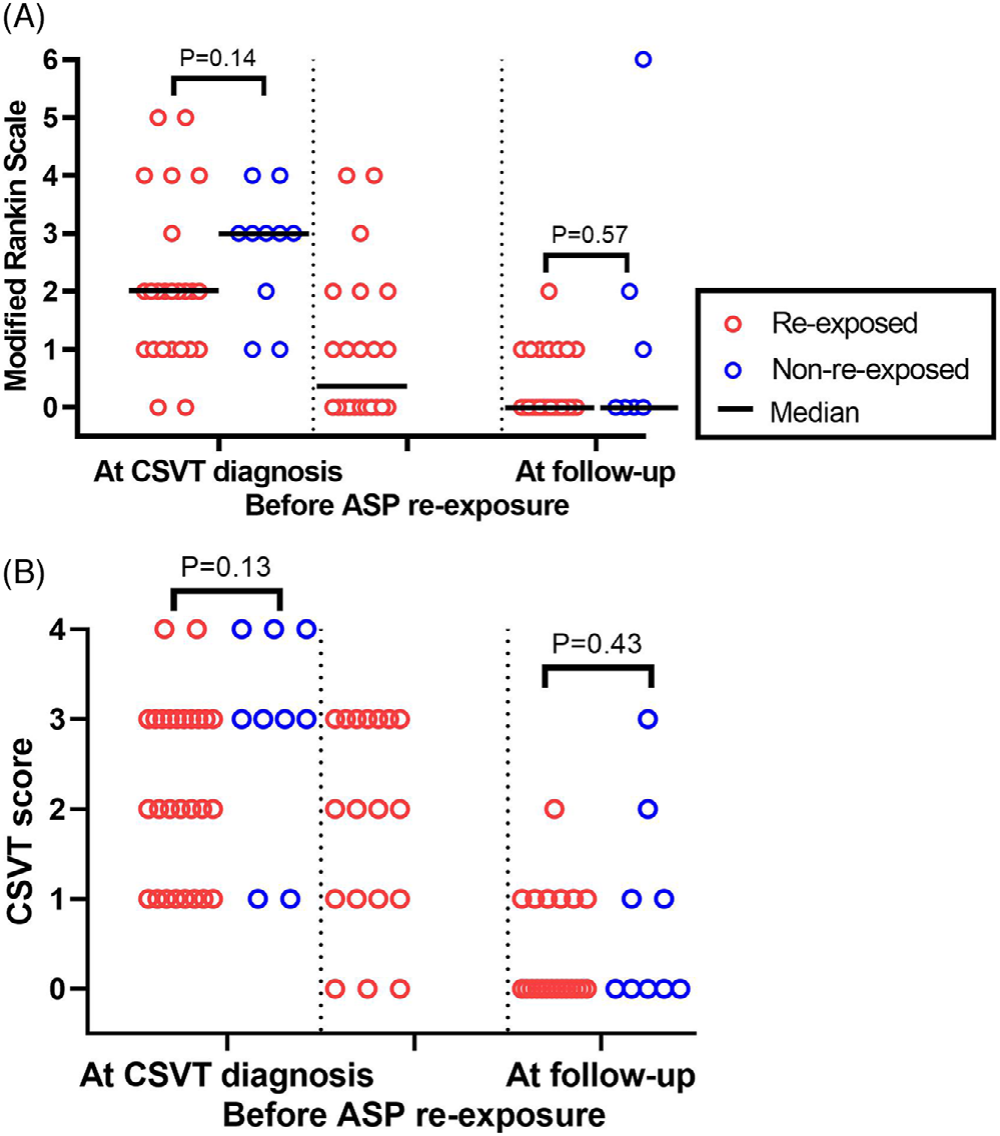
Neurological status (A) and thrombosis extent by imaging (B). (A) The modified Rankin Scale from 0 to 6 at three timepoints and (B) cerebral sinovenous thrombosis (CSVT) score quantified the extent of the CSVT

**FIGURE 3:**
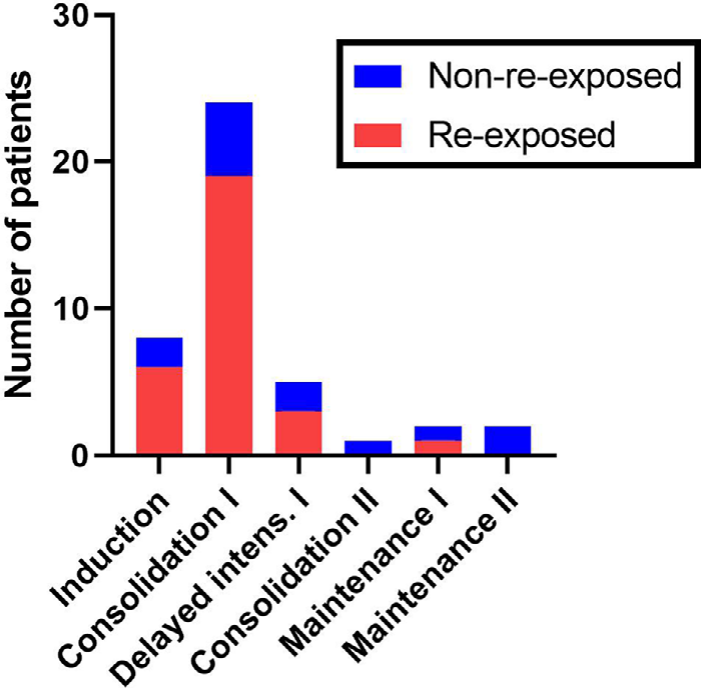
Treatment phase at cerebral sinovenous thrombosis (CSVT) diagnosis. Patients with CSVT during high‐risk blocks (*n *= 4) are not shown in the figure

All cases of CSVT (*n *= 46) were symptomatic, and symptoms at CSVT diagnosis were often reported concurrently (Table 3). At CSVT diagnosis, patients had a median mRS score of 2 (slight disability) (IQR: 1–4) (Figure 2A and Table 3). CSVT was confirmed in all patients, with either magnetic resonance imaging angiography (70%, *n* = 32), computed tomography angiography (28%, *n* = 13) or autopsy (2%, *n* = 1). Detailed reports of imaging at CSVT diagnosis were available in 39 patients (78%). The most frequent locations of thrombosis were the superior sagittal sinus (*n* = 33), sinus transversus (*n* = 20), sinus rectus (*n* = 5) and the confluence of sinuses (*n* = 4). The extent of thrombosis in cerebral veins and sinuses at CSVT diagnosis was comparable in later ASP re‐exposed and non‐re‐exposed patients (*p* = 0.13) (Table 3 and Figure 2B). Parenchymal lesions were reported in 16 patients, including oedema (*n* = 8), bleeding (*n* = 11) and one or more infarctions (*n* = 8). Thrombophilia was confirmed in four of 32 screened patients (prothrombin factor II heterozygosity [*n* = 2], factor V Leiden heterozygosity [*n* = 2]). CSVT occurred most frequently during induction before the start of ASP (17.4%, *n* = 8) and consolidation treatment (52.2%, *n* = 24). Four patients (8.7%) had CSVT during high‐risk (HR) blocks (Figure 3). At CSVT diagnosis, patients had received a mean of 2.7 ASP doses, all PEG‐ASP. In two‐thirds of patients (63%, *n* = 29), CSVT occurred within 14 days of steroid administration (Table 2). One adult patient received thromboprophylaxis at CSVT diagnosis, and one adult had a previous non‐cerebral TE at the time of CSVT.

### 3.3 Re‐exposure to ASP

Four patients were excluded from the re‐exposure analysis (Figure 1). Of the remaining 42 CSVT patients, 31 patients (74%) were re‐exposed to ASP at a median of 31 days (IQR: 21–57 days) after CSVT. All ASP doses after CSVT were administered during anticoagulation coverage. Patients re‐exposed to ASP were significantly earlier in their course of ALL treatment than non‐re‐exposed (median 50 vs. 81 days; *p* = 0.03) (Table 2 and Figure S1). In concordance, re‐exposed patients lacked significantly more ASP doses than non‐re‐exposed patients at CSVT diagnosis (mean 11.2 vs. 8.4 doses; *p* = 0.04). The patient who experienced a TE prior to CSVT was not re‐exposed to ASP. No significant difference was found between re‐exposed and non‐re‐exposed patients regarding age, sex, BMI, immunophenotype, presence of CNS leukaemia at ALL diagnosis, or ALL risk group at day 29 (Table 2). The prevalence of neurologic symptoms at CSVT diagnosis, including affected consciousness, did not differ between patients re‐exposed and non‐re‐exposed to ASP (Table 3). The grade of parenchymal lesions and CSVT score did not impact re‐exposure to ASP (Table 3 and Figure 2B). Patients with thrombophilia were re‐exposed to ASP. The neurological symptoms before ASP re‐exposure were available in 27 of 31 re‐exposed patients. The neurological status improved in the time between the diagnosis of CSVT diagnosis and ASP re‐exposure; mRS became normal in 50% (*n* = 11), being reduced from 2.0 to 0.5 (*p* < 0.01) (Figure 2A). A detailed description of imaging before re‐exposure was available in 23 of 31 cases a median of 22 days after CSVT. Imaging revealed partial recanalisation in at least one sinus in 16 patients (70%), complete recanalisation in all occluded sinuses in three (13%), no recanalisation in three (13%, *n *= 3), and progression of thrombosis in one patient (4%). The extent of CSVT evaluated by the CSVT score was reduced from grade III at CSVT diagnosis to grade II before ASP re‐exposure (*p* < 0.01) (Figure 2B).

### 3.4 CSVT‐related outcome

#### 3.4.1 Mortality, haemorrhages and re‐thrombosis

The median duration of follow‐up for CSVT patients was 4.5 years (IQR: 2.8–7.4) after CSVT diagnosis. All‐cause mortality in CSVT patients was 6.5% (95% CI: 1.4%–17.9%, *n* = 3). Two patients died due to CSVT, corresponding to a case fatality directly attributable to CSVT of 4.3% (95% CI: 0.5%–14.8%) (see Supporting Information for details). All patients received low‐molecular‐weight heparin after CSVT for a median of 232 days (IQR: 183–403). Four of 46 patients experienced major bleeding (cerebral and/or intraperitoneal, between 2 and 5 days after CSVT diagnosis) during antithrombotic treatment (bleeding rate of 8.7%, corresponding to an incidence rate of 0.1 per year during antithrombotic treatment) (Table 3). Two ALL relapses occurred in both the re‐exposed and non‐re‐exposed CSVT patients. Two of 31 patients re‐exposed to ASP (6.5%) experienced a re‐thrombosis. One patient had a second CSVT 82 days after the first CSVT (15 days after ASP re‐exposure) and fully recovered, with an mRS of 0 at the last follow‐up. The second patient with multiple thromboses at CSVT diagnosis (pulmonary embolism and bilateral deep vein thrombosis [DVT]) developed a re‐DVT 122 days after CSVT and 120 days after ASP re‐exposure.

#### 3.4.2 Impact on ALL treatment

The median number of omitted ASP doses per CSVT patient was 2.5 (IQR: 1–6.75), with significantly more omitted doses in non‐re‐exposed patients (median 6, IQR: 4–8) than in re‐exposed patients (median 2, IQR: 1–5) (*p* < 0.01). Eight of 31 re‐exposed patients experienced other ASP‐related toxicities after ASP re‐exposure, such as hypersensitivity (*n* = 4), pancreatitis (*n* = 2), osteonecrosis (*n* = 1) and hyperglycaemia (*n* = 1). After CSVT, at least one scheduled intrathecal chemotherapy was omitted in one‐third of patients, and the dose of steroids was reduced in one patient (Table 3).

#### 3.4.3 Neurological outcome and recanalisation

No disability (mRS score 0) was reported at the last follow‐up in 61% (*n* = 28) of CSVT patients (median mRS = 0, IQR: 0–1), with no significant difference between re‐exposed and non‐re‐exposed patients (*p* = 0.57) (Figure 2A and Table 3). Detailed imaging data during follow‐up revealed partial recanalisation (40.0%, *n* = 14) and complete recanalisation (57.1%, *n* = 20) in 35 available patients after a median time of 0.5 years (IQR: 0.3–1.2) after CSVT diagnosis. The grade of recanalisation and CSVT score at the last follow‐up were similar among re‐exposed and non‐re‐exposed patients (*p* > 0.43) (Table 3 and Figure 2B).

## 4 DISCUSSION

This study is the largest study to date, reporting on 46 patients diagnosed with CSVT during ALL treatment in a cohort of 2653 children and young adults. The majority of our patients with CSVT (74%) were re‐exposed to ASP. Neurological outcomes, measured by mRS, along with recanalisation of CSVT were comparable between re‐exposed and non‐re‐exposed patients at the time of last follow‐up. We could not identify significant factors impacting re‐exposure to ASP except in the early ALL treatment phase at CSVT diagnosis.

Previously, Musgrave et al. [1] reported 43 CSVT cases in children and young adults treated according to the UKALL 2003 protocol. One or more ASP doses were omitted in 18 of 42 (43%) cases, and 16 (37%) patients were not re‐exposed to ASP after CSVT diagnosis [1]. A similar proportion of our patients were re‐exposed to ASP, although ≥1 ASP doses were omitted in a larger proportion of our patients (84%, *n* = 37). The study of Musgrave et al. [1] found that intrathecal chemotherapy was omitted in two of 42 patients (5%) compared with our findings of ≥1 doses of omitted intrathecal treatment in one‐third of cases. We have no protocol‐specific explanations for the difference, although a decision made case by case may play a role. Klaassen et al. [30] reported on 26 children with CSVT (Dutch Childhood Oncology Group ALL‐10 protocol) but provided no information on alterations on ASP or intrathecal treatment in cases with CSVT. In adults (*n* = 20) with ALL or lymphoblastic lymphoma, CNS thrombosis occurred despite thromboprophylaxis in 90% of cases [2]; only one patient was re‐exposed to ASP after CNS thrombosis. In our study, only one adult patient received antithrombotic prophylaxis, and eight of 11 adults were re‐exposed to ASP.

The AEA level indicates the effect of ASP. Our subanalysis of median AEA in paediatric CSVT cases compared with the childhood ALL population did not show an association with CSVT (*p* = 0.41). This is in concordance with several other studies investigating the association of AEA and TE [31, 32, 33].

After a median follow‐up of 4.5 years, 61% of CSVT patients in our study had no neurologic sequelae, evaluated by mRS. Klaassen et al. [30] described permanent disabilities in nine of 26 patients (35%) after a median follow‐up of 4.9 years, similar to findings in our CSVT population, while a late neurological morbidity of 12% (5/43 cases) was found by Musgrave et al. [1]. No previous reports from the NOPHO study group have included information on neurologic sequelae [8, 20].

A major strength of the present study is the population‐based design, with quarterly registrations of CSVT with a very high compliance rate [19] In addition, we obtained detailed case descriptions on 100% of CSVT cases at multiple timepoints from local paediatric oncologists and adult haematologists involved in patient care. Furthermore, all centres scrutinised patient data to check for unregistered CSVT cases. Another strength is the relatively large sample size of a rare treatment complication from a large cohort of 2653 ALL patients and a long follow‐up to detect permanent neurological impairment due to CSVT.

Limitations include the observational design, which meant that there was a lack of imaging data in non‐re‐exposed patients at the time of possible ASP re‐exposure and no information on the frequency of re‐thrombosis after CSVT diagnosis in non‐re‐exposed patients. Two ALL relapses occurred in both the re‐exposed and non‐re‐exposed CSVT patients, which precludes a meaningful survival analysis. The limited sample size calls for caution when interpreting statistical results.

Recommendations in the NOPHO ALL2008 protocol were to re‐expose to ASP after CSVT if CSVT symptoms had resolved and recanalisation of veins was revealed by imaging during anticoagulation cover [8, 13]. International experts on the matter suggest ASP re‐exposure following stabilisation of the acute TE and only under the cover of anticoagulation [34]; however, reports on the safety of re‐initiation of ASP are warranted [13, 34].

The major threat to a patient with truncated ASP is the increased risk of relapse [17]. Our study indicates that clinically recovered CSVT patients with improved cerebral imaging can safely be re‐exposed to ASP during anticoagulation coverage.

## AUTHOR CONTRIBUTIONS

Mette Tiedemann Skipper designed the study and wrote the study protocol; collected, analysed and interpreted the data; and wrote and edited the manuscript. Ruta Tuckuviene initiated and designed the study, contributed to the study protocol and data collection, and reviewed the manuscript. Kjeld Schmiegelow served as the primary investigator of the NOPHO ALL2008 protocol, initiated the study and edited the manuscript. Cecilie Utke Rank, Kirsten Brunsvig Jarvis, Petter Quist‐Paulsen, Susanna Ranta, Jonas Abrahamsson, Helene Hallböök and Ulla Waitiovaara‐Kautto served as primary investigators, collected data and edited the manuscript. Liv Andrés‐Jensen contributed to data collection and edited the manuscript. Birgitte Klug Albertsen and Line Stensig Lynggaard collected and analysed data, and edited the manuscript. Pasi Huttunen, Sonata Trakymiene and Ruta Semaskeviciene served as primary investigators, collected data, translated imaging descriptions and edited the manuscript.

## CONFLICTS OF INTEREST

Birgitte Klug Albertsen: Sponsor of the investigator‐initiated trial NOR‐GRASPALL2016. Speaker and/or Advisory Board Honoraria from Erytech (2020) and Servier (2021). Kirsten Brunsvig Jarvis: Honoraria from Bayer (2021). Sonata Trakymiene: Honoraria from Bayer HealthCare, Novo Nordisk, Octapharma, Roche, and Takeda, served on advisory board committees for Novo Nordisk and Roche. Remaining authors declare they have no conflicts of interest.

## ETHICS STATEMENT

The study was approved by relevant national or regional ethics committees, and the study was performed according to the Declaration of Helsinki. The NOPHO ALL2008 protocol was approved by the National Medicines Agencies in all participating countries and relevant national or regional ethics committees. Clinical Trial Registration: EudraCT 2008‐003235‐20 and 2011‐000908‐18 (Lithuania).

## PATIENT CONSENT STATEMENT

All study participants provided written informed consent.

## Supporting information



Supporting information
