# Supplementary material for: Cerebral sinovenous thrombosis and asparaginase re‐exposure in patients aged 1–45 years with acute lymphoblastic leukaemia: A NOPHO ALL2008 study
Source: EJHaem. 2022 Jun 24;3(3):754–63. doi: 10.1002/jha2.484 (PMC9422014; doi:10.1002/jha2.484)
Supplement: Supplementary file 1 — Supporting information [file JHA2-3-754-s001.docx]

# Supplementary material

# The NOPHO ALL2008 protocol

The NOPHO ALL2008 treatment protocol was described regarding the asparaginase (ASP) treatment in the main manuscript and elsewere.^1^ Stratification of patients was carried out on day 29 with reclassification on day 79, allocating patients into standard risk (SR), intermediate risk (IR) and high-risk (HR) according to the karyotype of the leukemic clone and the bone marrow minimal residual disease status.^1^

All non-HR patients received five doses of pegylated ASP (PEG-ASP) 1000 IU/m^2^ intramuscularly at two-week intervals during consolidation therapy. A randomized controlled clinical trial was performed after consolidation therapy randomizing non-HR patients to either a) *the standard arm*; 10 additional PEG-ASP doses with two-week intervals or b) *the experimental arm;* 3 additional PEG-ASP doses with six-week intervals. In 2016, the trial was closed and all non-HR cases were treated according to the experimental arm afterwards.^2^ HR patients received one dose of ASP in each block during seven to nine blocks of chemotherapy depending on minimal residual disease level and two doses during delayed intensification, unless the patient was eligible for bone marrow transplantation. Induction included prednisolone 60 mg/m^2^ daily for all patients, except from patients with T-cell ALL or white blood cell (WBC) count ≥100 × 10^9^/L who received dexamethasone 10 mg/m^2^ daily. Prophylactic intrathecal methotrexate according to age was administered to SR and IR patients after induction. SR patients received eight doses and IR patients received 17 doses. HR patients were scheduled for 20 triple intrathecal injections consisting of cytarabine, methotrexate and prednisolone succinate. Central nervous system (CNS) involvement of leukemia was defined as no blasts in cerebrospinal fluid on cytospin and no other signs of CNS-leukemia (CNS1), 0–5 cells/mL cerebrospinal fluid with blasts on cytospin and no other signs of CNS leukemia (CNS2) and >5 cells/mL cerebrospinal fluid with blasts on cytospin, cranial nerve palsy, intracranial “leukemic” mass on MRI, eye involvement confirmed by MRI, or a biopsy to confirm CNS ALL (CNS3).^3^ Asparaginase enzyme activity (AEA) was measured systematically and levels of AEA was analyzed as previously described.^4^

# Asparaginase enzyme activity

## Methods/statistics

Asparaginase enzyme activity (AEA) was measured systematically in all children as part of the treatment protocol. Median AEA of each patient was included if at least two samples were available drawn 14 +/- 2 days from last ASP administration and the median AEA was above zero. Median AEA were not adjusted for number of AEA samples in each patient. Sufficient AEA was defined as AEA ≥100 IU/L within 14 days (+/- 2 days) after ASP administration.26 We compared median AEA between children in the CSVT population with children without TE diagnosed with ALL until March 2016 (the comparison population).

## Results

Median AEA was available in 20 children with CSVT. For the comparison population median AEA was available in 1062 children. Median AEA in the children with CSVT was 188 IU/l (95% CI: 142–266) and 210.5 IU/l (95% CI: 204–221) in the comparison population, p=0.41. AEA measurements were performed in 35 cases (76%) of children and adults with CSVT, of whom 19 (54%) had sufficient AEA (≥100 IU/ml), 9 patients (26%) had no valid samples available, four patients (11%) had no AEA (measured 1, 7, 12 and 14 days from ASP administration), and three patients (9%) had AEA <100 IU/ml, suggesting antibody mediated ASP inactivation. Of note, clinicians were not informed of the AEA status of the patient, thus not being used in the clinical decision-making of re-exposure. AEA ≥100 IU/ml was detected in 12 of 31patients who were re-exposed and seven of 11 who were non-re-exposed. Four of six patients with low or no AEA were re-exposed to ASP. Four patients (9%) developed ASP hypersensitivity after ASP re-exposure, one with no AEA and three with no valid samples.

# Fatal cases and major bleedings

Two of 46 patients died due to the CSVT, case A and B. Case A: The causes of death were cerebral herniation caused by hemorrhage at the time of CSVT on 11. day after CSVT diagnosis. Case B: CSVT diagnosed postmortem.

Four of 46 patients experienced major bleedings during antithrombotic treatment between two and five days after CSVT diagnosis. Two of the patients with major bleedings died because of the bleeding. Case A was mentioned above. Case C died due to intraabdominal bleeding caused by typhlitis and intestinal perforation, 4 days after CSVT diagnosis.

Three additional patients died during or after last follow-up due to second malignant neoplasm and bone marrow transplantation (2.3 years after CSVT, n=1), toxicities after bone marrow transplantation (1.6 years after CSVT, n=1), relapse (5.0 years after CSVT, n=1).

# Time from ALL diagnosis to CSVT diagnosis

The median time from ALL diagnosis to CSVT diagnosis were 53 days (50 days for patients re-exposed to ASP and 81 for patients non-re-exposed to ASP). The distribution of days from ALL diagnosis to CSVT diagnosis for both groups of patients is depicted in Figure S1 below. One patient in the non-re-exposed group is not shown since the patient had diagnosis of CSVT 799 days after ALL diagnosis.

**Figure S1**

# References:

1. Toft N, Birgens H, Abrahamsson J, et al. Risk group assignment differs for children and adults 1-45 yr with acute lymphoblastic leukemia treated by the NOPHO ALL-2008 protocol. *Eur. J. Haematol.* 2013;90(5):404–412.

2. Albertsen BK, Grell K, Abrahamsson J, et al. Intermittent Versus Continuous PEG-Asparaginase to Reduce Asparaginase-Associated Toxicities: A NOPHO ALL2008 Randomized Study. *J. Clin. Oncol.* 2019;37(19):1638–1646.

3. Rank CU, Toft N, Tuckuviene R, et al. Thromboembolism in acute lymphoblastic leukemia: Results of nopho all2008 protocol treatment in patients aged 1 to 45 years. *Blood*. 2018;131(22):2475–2484.

4. Tram Henriksen L, Gottschalk Højfeldt S, Schmiegelow K, et al. Prolonged first-line PEG-asparaginase treatment in pediatric acute lymphoblastic leukemia in the NOPHO ALL2008 protocol-Pharmacokinetics and antibody formation. *Pediatr. Blood Cancer*. 2017;64(12):.
